# Striae Gravidarum, Acne, Facial Spots, and Hair Disorders: Risk Factors in a Study with 1284 Puerperal Patients

**DOI:** 10.1155/2020/8036109

**Published:** 2020-05-19

**Authors:** Isadora da Rosa Hoefel, Magda Blessmann Weber, Ana Paula Dornelles Manzoni, Bárbara Hartung Lovato, Renan Rangel Bonamigo

**Affiliations:** ^1^Program of Pathology, Universidade Federal de Ciências da Saúde de Porto Alegre, Porto Alegre, Brazil; ^2^Dermatology Service of Santa Casa de Porto Alegre, Porto Alegre, Brazil; ^3^Faculdade de Medicina de Jundiaí, Brazil; ^4^Dermatology Service of Hospital de Clínicas de Porto Alegre, Universidade Federal do Rio Grande do Sul, Porto Alegre, Brazil

## Abstract

**Objective:**

To determine the prevalence of skin changes during pregnancy and to relate their occurrence to specific factors in a population of south Brazil.

**Methods:**

A cross-sectional analytical study was carried out with 1284 puerperal patients. A questionnaire about skin changes during pregnancy was developed and applied by the authors to all puerperal women admitted in a tertiary hospital in south Brazil.

**Results:**

The appearance of striae during pregnancy was reported by 633 women (49.5%) and had a statistically significant association with primiparity, presence of stretch marks before pregnancy, and gestational weight gain above 21 kg. Facial blemishes were reported by 33.9% (*n* = 434) and were associated with a positive family history, multiparity, and the use of facial sunscreen (*p* < 0.0001). The onset or worsening of acne was identified in 35.7% (*n* = 456) and was statistically associated with primiparity and Fitzpatrick phototypes IV and V. Hair alterations were reported by 44.5% (*n* = 569) and were associated with primiparity (*p* = 0.029).

**Conclusion:**

Although most of the skin changes during pregnancy are considered “physiologic,” they can cause significant discomfort. Thus, it is important to know them and to understand which risk factors may be associated with such changes.

## 1. Introduction

Pregnancy influences virtually all the maternal organic systems, which undergo significant modifications to allow retention and intrauterine development of the fetus. During pregnancy, the female body undergoes numerous hormonal, metabolic, immunological, and vascular changes [1].

In the skin and mucous membranes, pregnancy causes physiological changes, which can be divided into pigment alterations, hair alterations, nail alterations, skin gland alterations, and vascular alterations [2]. Many of these occur due to increased endocrine activity, in particular by increased production of the hormones progesterone and estrogen [3]. Although rare, there are also specific diseases of gestation, and the most common are pruritus of pregnancy, pemphigoid gestationis or herpes gestationis, polymorphic dermatitis of pregnancy, and impetigo herpetiformis [4]. In addition, autoimmune skin diseases often worsen during pregnancy, mainly systemic lupus erythematosus, dermatomyositis, and pemphigus [5].

Considering the multiplicity of physiological skin alterations that occur during pregnancy and the stigma they generate, few studies have attempted to analyze the epidemiological aspects related to the subject, something which could facilitate better management of such problems [6, 7]. Although physiological, these alterations can persist long after the gestational period and have a considerable impact on the patients' quality of life [3].

## 2. Materials and Methods

After approval by the Research Ethics Committee of the institution, a cross-sectional study was carried out, the objective of which was to identify the prevalence of the main skin alterations that occur during pregnancy and to relate their occurrence to specific factors.

The sample consisted of puerperal women hospitalized at the Mário Totta Maternity of Santa Casa Hospital (Porto Alegre, Brazil), during eight months (winter and spring).

All the admitted mothers (mothers of live newborns) who accepted to participate and signed the free and informed consent term were included in the study.

Data were collected using standardized questionnaires applied by four medical students and one dermatologist who jointly received training to standardize the interview. All the participants were interviewed on the first day after delivery, and data were collected on obstetric history, phenotypic characteristics, skin alterations developed during pregnancy, and skin care in pregnancy. The Fitzpatrick scale was used to determine the skin type of the participants [8].

The results are presented using descriptive statistics—absolute and relative distribution—as well as by measures of central tendency and variability, while the study of the distribution of age data was conducted using the Kolmogorov-Smirnov test. For the bivariate analysis between categorical variables, Pearson's chi-squared test (*χ*^2^) was used, and in the contingency tables in which at least 25% of the values presented an expected frequency of less than 5, Fisher's exact test was adopted. In situations where at least one variable had a polyatomic characteristic, the Monte Carlo simulation was used. For the continuous variables, when the comparison was made between two independent groups, the Student *t-* and the Mann-Whitney tests (asymmetric distribution) were applied. The data were analyzed in the Statistical Package for Social Sciences version 17.0 (SPSS Inc., Chicago, IL, USA, 2008) program for Windows, and for the statistical decision criteria, a significance level of 5% was adopted.

## 3. Results

The results presented refer to a sample of 1284 patients aged from 13 to 51 years, the mean being 26.6 (±6.8) years. The patients' general characteristics are presented in Table 1.

Multiparous patients represented 52.6% (*n* = 676), and two pregnancies were the median in this group.

Prior to pregnancy, the mean weight was 66.9 ± 15.7, and 39.3% (*n* = 496) of the patients gained up to 10 kg; 32.7% (*n* = 413) from 11 to 15 kg, and 10.7% (*n* = 135) gained more than 21 kg.

The prevalence of health problems during pregnancy was 48.7% (*n* = 624) within the sample, with the most common conditions being urinary infection (50.3%) (*n* = 314) and increased blood pressure (27.9%) (*n* = 174), while 87.3% (*n* = 1114) of the investigated patients used some type of medication and, in this group, 66.4% (*n* = 740) used ferrous sulfate; 33.5% (*n* = 373) used folic acid; and 30.3% (*n* = 338) reported the use of antibiotics (Table 1). Of the sample, 80.2% (*n* = 1024) had appropriate prenatal follow-up, considering a minimum number of 6 visits.

The daily use of some type of moisturizer was confirmed by 55.3% (*n* = 710) of those investigated, and the daily use of facial sunscreen during pregnancy was reported by 18.0% (*n* = 230) (Table 1).

The main skin changes that occurred in the recent pregnancy were stretch marks (49.5%, *n* = 633), facial blemishes (33.9%, *n* = 434), acne (35.7%, *n* = 456), and hair alterations (44.5%, *n* = 569). The important details of these alterations are described in Tables 2 and 3.

When assessing the relationship between age group and alterations, there was significant association of the up to 25-year age group with the appearance of stretch marks (66.3%; *n* = 402, *p* < 0.001), acne (*p* < 0.001), and the absence of facial blemishes (75.7%; *n* = 458, *p* < 0.0001). In the over 26-year age group, there was an association with the absence of stretch marks (65.8%; *n* = 443, *p* < 0.001), the presence of blemishes (42.6%; *n* = 287, *p* < 0.001), and nonappearance/nonworsening of acne (71.4%; *n* = 480, *p* < 0.001) (Tables 2 and 3).

## 4. Discussion

This research was carried out in a tertiary and university hospital (Santa Casa de Porto Alegre/Universidade Federal de Ciências da Saúde de Porto Alegre). This hospital receives patients from various parts of Greater Porto Alegre, most of whom received prenatal care in low-risk primary services, so that our sample resembles the population found in primary care settings. The demographic profile observed is very similar to that found in a study carried out among pregnant women in a primary healthcare unit in Porto Alegre: in both studies, the predominant age of interviewees was 20 to 29 years (46.9% versus 51.7%) and the main pathologies presented during pregnancy were urinary tract infections and arterial hypertension [9]. The weight gain observed in our sample, in which the predominant increase was up to 15 kg, is in line with the recommendations of the Ministry of Health in its Technical Manual for Prenatal and Puerperium [10]. Adequate prenatal care was performed by 80.2% of the pregnant women, considering a minimum of 6 consultations also recommended by the Ministry of Health. Regarding skin changes, the main considerations are described below.

In our study, 49.5% of the interviewees reported the appearance of stretch marks during pregnancy, a lower percentage than that found in the Brazilian and international literature, with values between 55 and 61% [11, 12]. In accordance with the literature, the main sites affected were, in descending order, abdomen, breasts, and thighs, and there was a statistically significant association between greater weight gain (>16 kg) and the development of stretch marks [11, 12]. In the present study, primiparity, excess weight gain (greater than 21 kg), the presence of stretch marks prior to the first pregnancy, and younger maternal age were found to be factors associated with the appearance of stretch marks. These data are consistent with those in the literature [13, 14].

The use of moisturizers and oils does not seem to have a preventive capacity for stretch marks during pregnancy, which has also been reported in a recent study published by Cochrane [15].

In this study, it was decided to include the occurrence of facial blemishes globally, not just melasma, since some pregnant women develop diffuse hyperpigmentation of the skin, the appearance or darkening of ephelides, and solar melanoses, which are different conditions of melasma, but which are still capable of causing discomfort in pregnant women.

The occurrence of facial blemishes during pregnancy was reported by 33.9% of the interviewees. Data on the occurrence of melasma and other spots on the face during pregnancy are quite heterogeneous in the literature, ranging from 10.7 to 70% [4, 16–18].

The factors associated with the appearance of facial blemishes in our study were family history of facial blemishes, multiparity, and the daily use of sunscreen on the face.

Although studies indicate a high prevalence of family history among women with melasma (ranging from 36 to 56.3%), few studies have been able to demonstrate a statistical association between family history and the development of melasma [7, 16–18]. The group that perceived the presence of facial blemishes had a significantly higher mean number of pregnancies when compared to those that did not present facial blemishes, corroborating data from the literature that associate the appearance of facial blemishes with increased parity [16, 18, 19].

In this study, the women who developed facial blemishes showed greater adherence to the daily use of facial sunscreen than those who did not develop such blemishes. Despite the known preventive and therapeutic action of the use of sunscreen in melasma, previous studies among pregnant women found no association between melasma prevention and sunscreen use [16, 17]. This is probably due to a reverse causality bias: women who are more likely to have melasma (e.g., family history or prior history of that skin alteration) are more likely to use sunscreen daily.

The literature is inconclusive regarding any association between the occurrence of melasma and facial blemishes and ethnicity or phototype: while some studies associate the occurrence of melasma with higher phototypes, others demonstrate no such association [16, 17]. In our study, no relationship was found between the occurrence of melasma and phototype.

The onset or worsening of acne lesions during pregnancy was reported by 35.7% of the interviewees, which could be related to the increase in glandular activity, already described in the literature, especially that of the sebaceous glands [20, 21].

Few studies, either Brazilian or international, address the development of acne in pregnancy. A study conducted in basic health units in São Paulo with a total of 124 pregnant women showed an incidence of 12.8% of acne lesions during pregnancy, and an Indian study with 607 pregnant women showed a prevalence of 2.3% among the women interviewed [20, 22].

In our sample, the factors associated with development or worsening of acne lesions during pregnancy were primiparity and maternal age less than 25 years. In a study carried out in Brazil with female patients with acne, the mean age of the patients was 21.7 years, which reinforces the data found in our study [23].

Phototypes 4 and 5 were also associated with a higher occurrence of acne in the present study. Interestingly, a recent study carried out in Pelotas (southern Brazil) found that patients with higher phototypes have a different pattern of acne than lighter-skinned patients, with noninflammatory acne prevailing in the former and inflammatory acne prevailing in the latter [24]. New studies into the occurrence of acne in the different phototypes could be conducted, as well as into the risk factors for and protection against the development of acne during pregnancy.

The occurrence of hair alterations during pregnancy was reported by 44.5% of the sample, with most complaints referring to hair loss and dryness. The data available in the literature show much lower rates of capillary changes during pregnancy, ranging from 2.6 to 12.8%, with both hair loss and increasing hair volume [7, 22].

The present data do not corroborate some studies that point to increased capillary volume in pregnancy (with increased thread diameter and a greater proportion of anagen to telogen threads) [25].

There was a greater proportion of capillary alterations among the primiparous patients, suggesting that perhaps the first pregnancy influenced the capillary cycle more strongly, or even a bias of confusion and memory, as women in their first pregnancy could be more aware of such modifications. In agreement with the literature, no other risk or protection factors for capillary alterations during pregnancy were identified.

Our study has limitations. Additional sample variables, such as weight and sex of the newborn, delivery route, gestational age, and economic and educational factors of the sample could have been collected and analyzed in order to enrich our analysis. Some of our data were only obtained through patient reports, such as family history of facial blemishes and the occurrence of hair alterations, which makes our data subject to biases of subjectivity and memory. The occurrence of other pigmentary alterations, such as *linea nigra*, and vascular alterations, such as palmar erythema, has not been studied (such changes are frequent but usually spontaneously resolved).

## 5. Conclusion

Given their high prevalence rates, the importance of skin alterations during pregnancy is clear. In particular, stretch marks, hair alterations, acne, and facial blemishes were observed.

Risk factors were found, and the recognition of these associations may help in the prevention and management of the problems. The main points are as follows: excessive weight gain, primiparity, and younger age as risk factors for stretch marks; the lack of evidence of the use of topical preparations during pregnancy to prevent stretch marks; family history, multiparity, and older age as risk factors for the development of facial blemishes; primiparity and the younger age as a risk factor for acne; and primiparity as a risk factor for hair loss and hair dryness.

Thus, the present study presents important data from a large sample, the largest Brazilian series on the subject, to date.

## Figures and Tables

**Table 1:** General characteristics, skin care, and skin changes during pregnancy. Santa Casa Hospital, Porto Alegre, Brazil.

Variables	Sample (*n* = 1284)	
	*n*	%
Age (years)		
Mean ± SD	26.6 ± 6.8	
Median (min–max)	26.0 (13-51)	
Age group		
≤19 years	229	17.8
20 to 29 years	622	48.5
30 to 39 years	397	30.9
≥40 years	35	2.7
Primiparous		
No	676	52.6
Yes	608	47.4
Number of pregnancies		
Mean ± SD	2.1 ± 1.5	
Median (min–max)	2.0 (1.0–7.0)	
Gestation		
1	569	44.4
2	372	29.0
3	175	13.7
4	81	6.3
5 or more	85	6.6
Phototype		
1	14	1.1
2	250	19.6
3	582	45.6
4	322	25.2
5	100	7.8
6	8	0.6
Daily moisturizing products use		
No	572	44.6
Yes	710	55.3
Type		
Moisturizing lotions	494	69.6
Ointments	323	45.5
Others	2	0.3
Weigh before pregnancy		
Mean ± SD	66.9 ± 15.7	
Median (min–max)	64.0 (68.0–88.0)	
Weigh after pregnancy		
Mean ± SD	79.4 ± 15.4	
Median (min–max)	78.0 (68.0–88.0)	
*p ^¶^*	0.0001	
Weight gain		
≤10 kg	496	39.3
11 to 15 kg	413	32.7
16 to 20 kg	219	17.3
≥21 kg	135	10.7
Stretch marks prior to first pregnancy		
No	775	60.6
Yes	503	39.4
Appearance of stretch marks during pregnancy		
No	647	50.5
Yes	633	49.5
Abdomen	514	81.2
Breasts	131	20.7
Flank	18	2.8
Thighs	113	17.9
Gluteus	85	13.4
Appearance or aggravation of acne		
No	820	64.3
Yes	456	35.7
Face	405	88.8
Back	125	27.4
Chest	54	11.8
Daily facial sunscreen use		
No	1047	82.0
Yes	230	18.0
Mother or sister diagnosed with facial blemishes/melasma		
No	811	63.3
Yes	430	33.6
Unknown	40	3.1
Appearance of facial blemishes/melasma		
No	845	66.1
Yes	434	33.9
Developed health complications during pregnancy		
No	657	51.3
Yes	624	48.7
Arterial hypertension	174	27.9
Diabetes	100	16.0
Urinary tract infection	314	50.3
Others	138	22.1
Medication use		
No	162	12.7
Yes	1114	87.3
Antibiotics	338	30.3
Iron sulfate	740	66.4
Folic acid	373	33.5
Antispasmodic	34	3.1
Others	410	36.8
Hair alterations		
No	709	55.4
Yes	569	44.5
Hair loss	166	29.2
Faster hair growth	103	18.1
Dry hair	111	19.5
Previous health conditions		
No	996	78.0
Yes	281	22.0
Prenatal care		
No	23	1.8
Yes	1258	98.2
Prenatal visits		
Mean ± SD	8.6 ± 3.5	
Median (min–max)	9.0 (0.0-40.0)	
Adequate prenatal care		
No	253	19.8
Yes	1024	80.2

^¶^Student's *t*-tests. SD: standard deviation; min: minimum; max: maximum.

**Table 2:** Stretch marks and facial blemishes in pregnancy. Santa Casa Hospital, Porto Alegre, Brazil.

Variables	Appearance of stretch marks^a^				*p* ^§^
	0—no (*n* = 647)		1—yes (*n* = 633)		
	*n*	%	*n*	%	

Age range					<0.001
<25 years	204	33.7	402	66.3	
26 years or more	443	65.8	230	34.2	
Stretch marks prior to first pregnancy					0.035
No	410	63.5	363	57.7	
Yes	236	36.5	266	42.3	
Primiparous					<0.0001
No	411	63.5	264	41.7	
Yes	236	36.5	369	58.3	
Phototype					0.218
1	7	1.1	7	1.1	
2	118	18.3	132	21.0	
3	314	48.8	265	42.2	
4	160	24.8	162	25.8	
5	41	6.4	58	9.2	
6	4	0.6	4	0.6	
Weight gain					<0.0001
≤10 kg	283	44.6	212	33.9	
11 to 15 kg	204	32.2	207	33.1	
16 to 20 kg	106	16.7	113	18.1	
≥21 kg	41	6.5	94	15.0	
Daily moisturizing products use					0.869
No	291	45.0	281	44.4	
Yes	355	54.9	352	55.6	
Adequate prenatal care					0.278
No	135	21.0	117	18.6	
Yes	508	79.0	513	81.4	

Variables	Appearance of facial spots/melasma^b^				*p* ^§^
	0—no (*n* = 845)		1—yes (*n* = 434)		
	*n*	%	*n*	%	

Age range					0.001
<25 years	458	75.7	147	24.3	
26 years or more	386	57.4	287	42.6	
Mother or sister diagnosed with facial blemishes/melasma					<0.0001
No	582	69.0	226	52.1	
Yes	230	27.3	200	46.1	
Unknown	31	3.7			
Primiparous					<0.0001
No	413	48.9	261	60.1	
Yes	432	51.1	173	39.9	
Phototype					0.902
1	10	1.2	4	0.9	
2	165	19.6	84	19.5	
3	386	45.9	196	45.6	
4	205	24.4	115	26.7	
5	69	8.2	29	6.7	
6	6	0.7	2	0.5	
Daily facial sunscreen use					<0.0001
No	724	86.2	319	73.7	
Yes	116	13.8	114	26.3	
Developed health complications during pregnancy					0.894
No	434	51.5	222	51.2	
Yes	498	48.5	212	48.8	
Number of pregnancies, groups					<0.001
1	416	49.2	151	34.8	
2	241	28.5	131	30.2	
3	100	11.8	76	17.5	
4	50	5.9	30	6.9	
5 or more	38	4.5	46	10.6	

^a^Percentages calculated based on the total of each group that noted the appearance of stretch marks. ^b^Percentages calculated based on the total of each group that noted the appearance of facial spots/melasma. ^§^Pearson's chi-squared test.

**Table 3:** Acne and hair abnormalities in pregnancy. Santa Casa Hospital, Porto Alegre, Brazil.

Variables	Appearance or aggravation of acne^a^				
	0—no (*n* = 820)		1—yes (*n* = 456)		*p*
	*n*	%	*n*	%	

Age range					<0.001^§^
<25 years	339	56.2	264	43.8	
26 years or more	480	71.4	192	28.6	
Primiparous					<0.001^§^
No	470	57.3	201	44.1	
Yes	350	42.7	255	55.9	
Phototype					0.011^§^
1	8	1.0	6	1.3	
2	154	18.8	96	21.2	
3	402	49.2	178	39.4	
4	198	24.2	123	27.2	
5	50	6.1	46	10.2	
6	5	0.6	3	0.7	
Weight gain					0.249^§^
≤10 kg	333	41.4	161	35.7	
11 to 15 kg	252	31.3	157	34.8	
16 to 20 kg	134	16.6	84	18.6	
≥21 kg	86	10.7	49	10.9	
Daily facial sunscreen use					0.272^§^
No	660	81.1	381	83.6	
Yes	154	18.9	75	16.4	
Developed health complications during pregnancy					0.944^§^
No	422	51.5	233	51.3	
Yes	397	48.5	221	48.7	
Medication use					
No	100	12.2	61	13.5	0.510^§^
Yes	717	87.8	390	86.5	

Variables	Appearance of hair alterations^b^				
	0—no (*n* = 709)		1—yes (*n* = 569)		*p*
	*n*	%	*n*	%	

Age range					0.374^§^
<25 years	343	56.7	261	54.4	
26 years or more	366	54.4	307	45.6	
Primiparous					0.029^§^
No	395	55.7	278	48.9	
Yes	314	44.3	291	51.1	
Phototype					0.533^¶^
1	7	1.0	7	1.2	
2	126	17.9	123	21.8	
3	326	46.2	254	45.0	
4	187	26.5	133	23.5	
5	56	7.9	43	7.6	
6	3	0.4	5	0.9	
Developed health complications during pregnancy					0.488^§^
No	370	52.3	285	50.2	
Yes	337	47.7	283	49.8	
Adequate prenatal care					
No	156	22.2	95	16.8	0.011^§^
Yes	548	77.8	472	83.2	

^a^Percentages calculated based on the total of each group that noted the appearance of aggravation of acne. ^b^Percentages calculated based on the total of each group that noted the appearance of hair abnormalities. ^§^Pearson's chi-squared test. ^¶^Fischer's exact test using Monte Carlo simulations.

## Data Availability

The data used to support the findings of this study are available from the corresponding author upon request.
